# Analysis of desertification combating needs based on potential vegetation NDVI—A case in the Hotan Oasis

**DOI:** 10.3389/fpls.2022.1036814

**Published:** 2022-12-14

**Authors:** Lei Zhang, Jia Qu, Dongwei Gui, Qi Liu, Zeeshan Ahmed, Yi Liu, Zhiming Qi

**Affiliations:** ^1^ State Key Laboratory of Desert and Oasis Ecology, Xinjiang Institute of Ecology and Geography, Chinese Academy of Sciences, Urumqi, Xinjiang, China; ^2^ Cele National Station of Observation and Research for Desert Grassland Ecosystem in Xinjiang, Cele, Xinjiang, China; ^3^ University of Cinese Academy of Sciences, Beijing, China; ^4^ Xinjiang University, Urumqi, Xinjiang, China; ^5^ McGill University, Department of Bioresource Engn, Saitne Anne De Bellevue, PQ, Canada

**Keywords:** desertification combating, potential natural vegetation, potential NDVI, over control, maxent

## Abstract

Combating desertification is vital for arresting land degradation and ensuring sustainable development of the global ecological environment. This study has analyzed the current desertification status and determined its control needs based on the difference between potential normalized difference vegetation index (PNDVI) and actual normalized difference vegetation index (ANDVI) in the Hotan desertoasis. The MaxEnt model, combined with the distribution point data of natural vegetation with long-term stable normalized difference vegetation index (NDVI) and 24 environmental factors was used to predict the PNDVI spatial distribution of different vegetation coverage grades and compared it with ANDVI. Excluding the areas of intense human activity such as arable land, the simulation results show that PNDVI with high, medium, and low vegetation cover was mainly distributed in the southwest and southeast of Hotan Oasis, in the midstream and downstream of Kalakash River and Yulong Kashi River, and the desert or Gobi area outside the oasis, respectively. The distribution of PNDVI with high, medium, and low vegetation cover accounted for 6.80%, 7.26%, and 9.17% of Hotan oasis, respectively. The comparison between ANDVI and PNDVI shows that 18.04% (ANDVI < PNDVI, about 3900 km^2^) of the study area is still suffering from desertification, which is mainly distributed in the desert-oasis ecotone in Hotan. The findings of this study implied that PNDVI could be used to assess the desertification status and endorsement of desertification control measures in vulnerable ecosystems. Hence, PNDVI can strengthen the desertification combating efforts at regional and global scales and may serve as a reference point for the policymakers and scientific community towards sustainable land development.

## Introduction

Desertification has been defined as the land degradation in arid, semi-arid, dry sub-humid areas (collectively known as dryland), resulting from various factors, including climate change and human activity (The United Nations Convention to Combat Desertification (UNCCD), 1994). Desertification has become a global challenge, causing unsustainable land management, and threatening the livelihoods of vulnerable populations (Barbier and Hochard, 2018; Cai et al., 2022). Anthropogenic climate change has driven over 5 million km2 of drylands toward desertification over the past three decades, affecting about 213 million people, 93% of them live in developing economies (Burrell et al., 2020). In addition, Global Network and Map 30 show that the gross change in Asia is 4.4 times larger than net change from 2000 to 2010 (Sbafizadeh-Moghadam et al., 2019). Therefore, governments and relevant institutes are trying to identify the relationship between human activity, variation of biophysical attributes of the landscape, and desertification to prevent and control desertification (Salvati et al., 2016). Failure of the UNCCD, aiming to reduce the rate of desertification, triggered the emergence of the land degradation neutrality (LDN) paradigm (Chasek et al., 2019). To achieve the target of LDN in 2030 and consolidate the desertification combatting efforts require precise identification of decertifying areas and site-specific desert prevention measures.

Given the far-reaching consequences of desertification, identification of desertification and relevant desertification control measures are still major challenges for the scientific community and policymakers (Dong et al., 2020; Hu et al., 2022). With the advancement of remote sensing technology, the difference between vegetation biophysical indexes obtained by remote sensing image inversion has been widely used in large-scale and long-term desertification evaluation and monitoring, such as the normalized difference vegetation index (NDVI) and the net productivity (NPP). NDVI derived from satellite data is an important vegetation indicator representing vegetation greenness, revealing the response of vegetation dynamics to the development of desertification (Zhou et al., 2014; Kalisa et al., 2019). NDVI has a positive correlation with NPP, which indicates vegetation growth status and ecosystem health (Bobee et al., 2012; Philippon et al., 2014; Elnashar et al., 2022). A decrease in vegetation cover decreases NDVI values reflecting an increase in desertification. Existing studies on NDVI for desertification assessment are static and better at warning than decision making. Moreover, objective measurement of desertification is difficult due to multiple criteria and the lack of a reliable baseline. At the same time, Minaei et al. (2018) showed that the role of climate and human-made interventions into the type and extent of land transformation is recommended in land degradation research. The theoretical benchmark of desertification needs to exclude the interference of human activities. Therefore, to properly assess desert-prone areas and relevant desert combating measures, we need to explore the maximum vegetation potential under current climate conditions, excluding human activity. This may serve the purpose of a baseline or benchmark to accurately identify the regions and extent of land degradation (Pan and Xu, 2020). Thus PNDVI, the optimum NDVI with an optimal climate and no human disturbance, can be used as a reference to measure degradation based on the difference between actual and potential NDVI (Stoms and Hargrove, 2000; Strandberg et al., 2022).

PNDVI reflects the growth of potential natural vegetation (PNV), a theoretical climax vegetation community that would occupy an area if there is no human interference (Paruelo and Lauenroth, 1995; Chytry, 1998). Unlike the original vegetation before man-made interference, the concept of PNV is to predict the final state of future development of vegetation in the region based on current vegetation (Zerbe, 1998). Exploration of potential vegetation without human disturbance is important to predict actual vegetation development under climate models. Therefore, the NDVI of PNV can be used as a real benchmark under current climate conditions to estimate the extent of desertification. The magnitude of the difference between actual and potential NDVI provides a quantitative measure of the overall the magnitude and pattern of land degradation and ecosystem functioning. However, limited studies on PNDVI warrant further exploration of this concept for the assessment of desertification extent and relevant prevention measures in drylands.

PNDVI is usually simulated based on the relationship between ANDVI and the natural environmental factors (Gao et al., 2012). At the end of the last century, Paruelo and Lauenroth (1995) have proved that the atmospheric and ecological processes can be linked interactively by empirical relationships between some traits of the NDVI curves and climate variables. Therefore, the relationship between NDVI data for natural areas and climate variables enables us to produce maps of the PNDVI. Classification and Regression Tree (CART) is the main model used to construct the quantitative relationship between ANDVI and climate, and to simulate the spatial distribution of PNDVI (Pan and Xu, 2020; Ma et al., 2021). However, with the development of technology and ecological niche theory, more and more statistical methods and software are used to construct the empirical relationship between vegetation and climate, such as surface range envelope (SRE) (Sormunen et al., 2011), multiple adaptive regression splines (MARS) (Elith and Leathwick, 2007), generalized boosting models (GBMs) (Heikkinen et al., 2012), random forests (RFs) (Barbet-Massin et al., 2013), flexible discriminant analysis (FDA) (Kuemmerlen et al., 2014), artificial neural networks (ANNs) (Ficko et al., 2011), generalized linear models (GLMs) (Mainali et al., 2015), and maximum entropy (MaxEnt) (Zhang et al., 2011). However, MaxEnt is the most widely used species distribution model at present, with numerous advantages, including ease of operation, short running time and high precision (Phillips et al., 2017). Furthermore, MaxEnt model is appropriate for the presence only data, which can simultaneously use continuous numerical or classified environmental factors as environmental data to participate in modeling and the operation is simple and the demand for sample size is small (Guo et al., 2019). Therefore, we attempt to use the MaxEnt model to simulate PNDVI.

An oasis is a unique ecological habitat for plants, humans, and wildlife in desert areas of northwest China. The sustainability of the oasis is highly important for smooth ecosystem functioning and stable economic development of the region. However, desertification due to harsh climate and overexploitation of resources by humans is seriously threatening the future development of oasis. Hotan Oasis located in the southern edge of Tarim Basin, with a complete desert oasis landscape structure. Therefore, combating desertification and maintaining the stability of the oasis ecosystem necessitate accurate identification of desert-prone areas and targeted control measures for sustainable development in arid regions. Hence, this study aims to quantify the current desertification status in Hotan oasis through calculation of PNDVI using MaxEnt model and to analyze the demand for desertification control needs in different zones of Hotan oasis by comparing the spatial distribution characteristics of PNDVI and ANDVI. The findings of this study may provide a reference point for researchers and policy makers to pin point desertification areas and implement targeted policy measures to halt desertification in arid regions.

## Study area

Hotan Oasis located in the southern margin of Tarim Basin and northern foot of the Kunlun Mountains of northwest China (Zhang et al., 2022). The Kalakash River and Yulong Kashi River originated in the Kunlun Mountains supply water to the Hotan oasis. Hotan oasis has typical continental desert climate characteristics such as warm and dry, abundant light and heat resources (Zhao et al., 2009). This oasis also falls into the category of continental warm temperate monsoon climate, with the average annual temperature being 13°C, annual precipitation being less than 50 mm, and evaporation over 2700 mm per year (Huang et al., 2022). At present, the ecological environment of Hotan Oasis is deteriorating due to long-term sandstorm disasters and large-scale development and utilization of water and soil resources in the basin (Yao et al., 2022).

## Materials and methods

### Data source and processing

**Figure 1:**
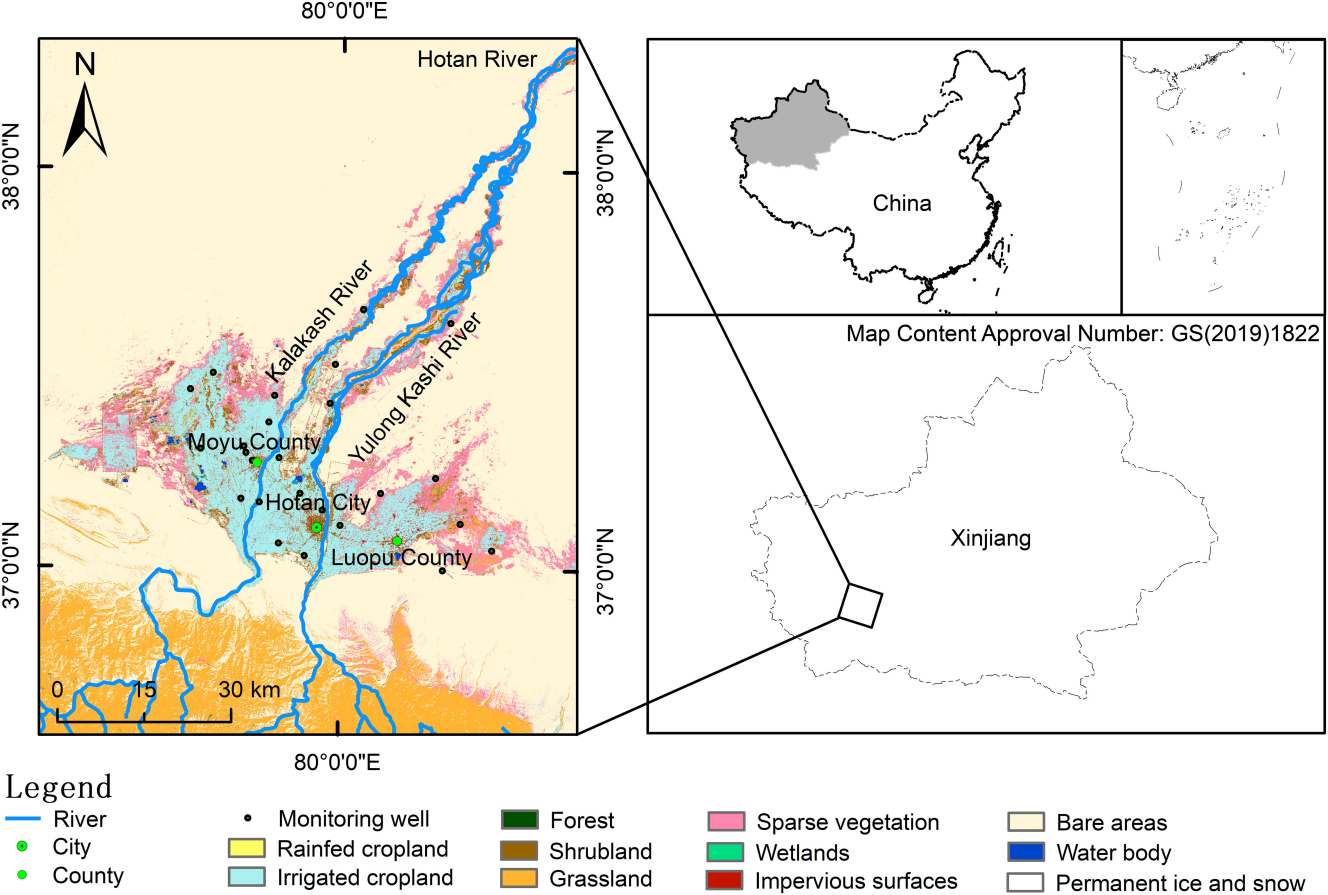
Location of the study area and Land-use type. Land-use type is based on the data from the Aerospace Information Research Institute, Chinese Academy of Sciences in 2020 (Zhang et al., 2021).

**Figure 2:**
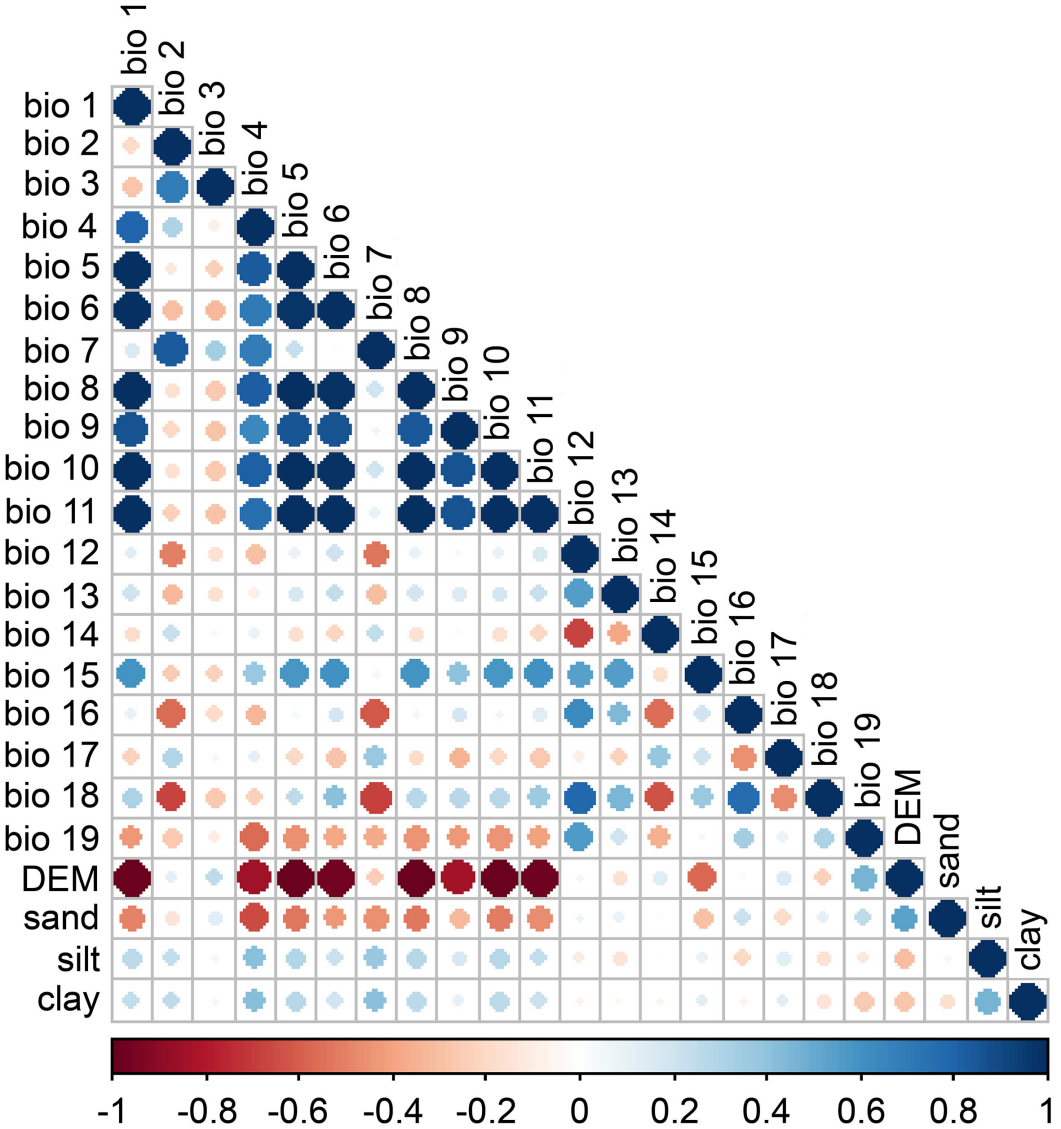
Correlation coefficients between the environmental factors. Positive correlations are shown in blue and negative correlations are shown in red. The color intensity and the size of the circle are proportional to the correlation coefficient.

In order to simulate the empirical relationship between ANDVI and climate variables and predict the geographical distribution of PNDVI, an environmental factor dataset that can characterize environmental characteristics must be defined (Lu et al., 2012). In this study, we used 24 environmental factors consisting of climate, soil, topography, and hydrology (**Table 1**). Bioclimatic variables were obtained from the WorldClim database (https://www.worldclim.org/), and the resolution was 30 s (≈1km^2^). Soil variables were downloaded from the SoilGrids (https://soilgrids.org), and the resolution was 250 m. The digital elevation model (DEM) data was downloaded from Geospatial Data Cloud (http://www.gscloud.cn), and the resolution was 250 m. Considering the importance of groundwater, we obtained the groundwater level of 25 observation wells (**Figure 1**) from Hotan Water Conservancy Bureau, and obtained the distribution data of groundwater level by inverse distance weighing method (Noori et al., 2013). The correlation between environmental variables can easily lead to multicollinear explanatory problems, such as increasing the variance of parameter estimation, and making the test of explicitness of variables meaningless. Therefore, this study eliminates the impact on the simulation results by multicollinearity test of 24 environmental variables (Graham, 2003). Firstly, the MaxEnt model was used to obtain the contribution rate of each variable, and the variables with contribution rate less than 1 are eliminated. Further, the Pearson correlation analysis of the data after modeling was carried out by ArcMap software (**Figure 2**). The variables with large contribution rate having correlation coefficient |r| > 0.8 were selected for subsequent model analysis (Yang et al., 2013). After the above screening process, environmental factors were finally selected for PNDVI prediction (**Table 2**).

**Table 1:** Description of bioclimatic variables used for MaxEnt model prediction.

Code	Environmental variables	Units
bio1	Annual Mean Temperature	°C
bio2	Mean Diurnal Range	°C
bio3	Isothermally (BIO2/BIO7) (* 100)	%
bio4	Temperature Seasonality (standard deviation *100)	%
bio5	Maximum Temperature of Warmest Month	°C
bio6	Minimum Temperature of Coldest Month	°C
bio7	Temperature Annual Range (Bio5-Bio6)	°C
bio8	Mean Temperature of Wettest Quarter	°C
bio9	Mean Temperature of Driest Quarter	°C
bio10	Mean Temperature of Warmest Quarter	°C
bio11	Mean Temperature of Coldest Quarter	°C
bio12	Annual Precipitation	mm
bio13	Precipitation of Wettest Period	mm
bio14	Precipitation of Driest Period	mm
bio15	Precipitation Seasonality (coefficient of variation)	%
bio16	Precipitation of Driest Quarter	mm
bio17	Precipitation of Wettest Quarter	mm
bio18	Precipitation of Warmest Quarter	mm
bio19	Precipitation of Coldest Quarter	mm
clay	Proportion of clay particles(<0.002mm) in the fine earth fraction	g/kg
sand	Proportion of sand particles (>0.05 mm) in the fine earth fraction	g/kg
silt	Proportion of silt particles (≥0.002 mm and ≤0.05mm) in the fine earth fraction	g/kg
DEM	digital elevation model	m
GWD	groundwater level in growing season	m

**Table 2:** The correspondence table between NDVI value and vegetation cover level.

NDVI		Vegetation coverage	Vegetation coverage level	Environmental factors	Contribution
NDVI_-1~1_	NDVI_0~255_				
>0.50	>191	>60%	High vegetation	boi11	24.7%
				boi18	14.7%
				boi19	7.9%
				bio3	5.4%
				clay	3.3%
				bio13	2.5%
				silt	2.4%
				bio2	1.3%
				bio15	1.1%
				GWD	1.0%
0.23~0.49	156~190	30%~60%	Medium vegetation	bio1	43.2%
				clay	7.7%
				bio2	5.4%
				bio18	4.6%
				bio19	2.7%
				bio3	2.7%
				bio16	1.8%
				silt	1.7%
				GWD	1.1%
0.09~0.22	139~155	15%~30%	Low vegetation	bio1	29.6%
				bio7	10.3%
				bio18	7.1%
				bio16	6.7%
				clay	2.5%
				bio4	1.4%
<0.08	<138	<15%	Bare or sparse vegetation	bio8	67.4%
				bio18	3.7%
				sand	2.8%
				clay	2.4%
				bio7	2.1%
				bio3	1.9%
				GWD	1.0%

**Figure 3:**
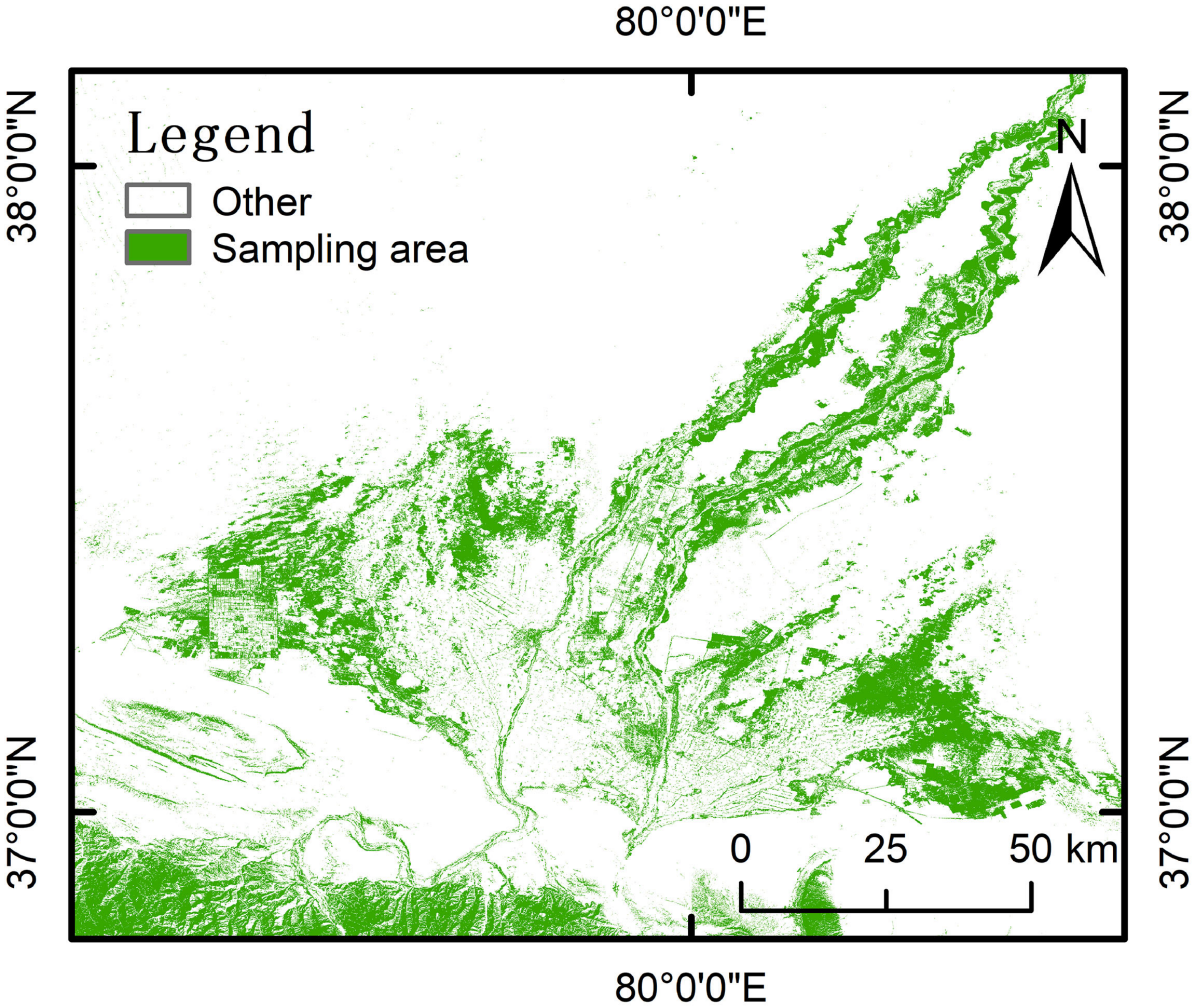
Sampling area.

In order to ensure that PNDVI simulation results are close to the natural state, modeling sampling points need to be extracted from natural vegetation areas where NDVI did not change significantly from 2005 to 2015.Therefore, NDVI and land cover data were used for point acquisition and model simulation. Land cover data in Hotan comes from the Aerospace Information Research Institute, Chinese Academy of Sciences (https://data.casearth.cn/), data resolution is 30 m (**Figure 1**). NDVI data for 2005 and 2015 were provided by National Ecosystem Science Data Center, National Science and Technology Infrastructure of China (http://www.nesdc.org.cn), and NDVI data for 2020 was calculated through Sentinel 2A image (https://scihub.copernicus.eu/). The steps to obtain the sampling area by ArcGIS 10.7 were as follows: (1) The threshold segmentation of NDVI was divided into four grades according to the classification method of Yao et al. (2022)’s study in temporal and spatial changes of vegetation cover in Hotan Oasis (**Table 2**); (2) natural vegetation (forest, shrub and grassland) extracted from land cover data by reclassification method; (3) NDVI with a stable value during 2005~2015 was calculated by raster calculator; (4) Extracting sampling area where appeared simultaneously in the results of (2) and (3) (**Figure 3**). To keep the consistency of spatial resolution and the minimum deformation of area, all data were projected to the GCS_WGS_1984 coordinate system and resampled to the 30 m resolution by ArcGIS 10.7.

### Methodology

MaxEnt (Maximum entropy model) was used to simulate the species distribution based on presence data (species presence) and environmental factor data (Phillips et al., 2006). The sorted distribution point data and the screened bioclimatic variable data were imported into MaxEnt 3.4.1 and the bioclimatic variables were evaluated by the Jackknife test (Merow et al., 2013). The models split the dataset by using 10-fold cross-validation, this method refers to randomly dividing the original dataset into 10 parts with nearly equal sample sizes, taking turns merging 9 of them as training set and the remaining 1 as test set. In practice, model performance was evaluated by calculating the Area Under the Receiver Operator Curve (AUC), its value ranges from 0.5 to 1. It is generally understood that AUC = 0.5 indicates the random distribution was indicated, AUC = 1 indicates that the predicted distribution area of the model was completely consistent with the actual distribution area of the research object, and prediction results can be considered satisfactory for our study when AUC > 0.7 (Phillips and Dudik, 2008).

## Results

### Contribution of main environmental factors to the simulation of PNDVI distribution

**Figure 4:**
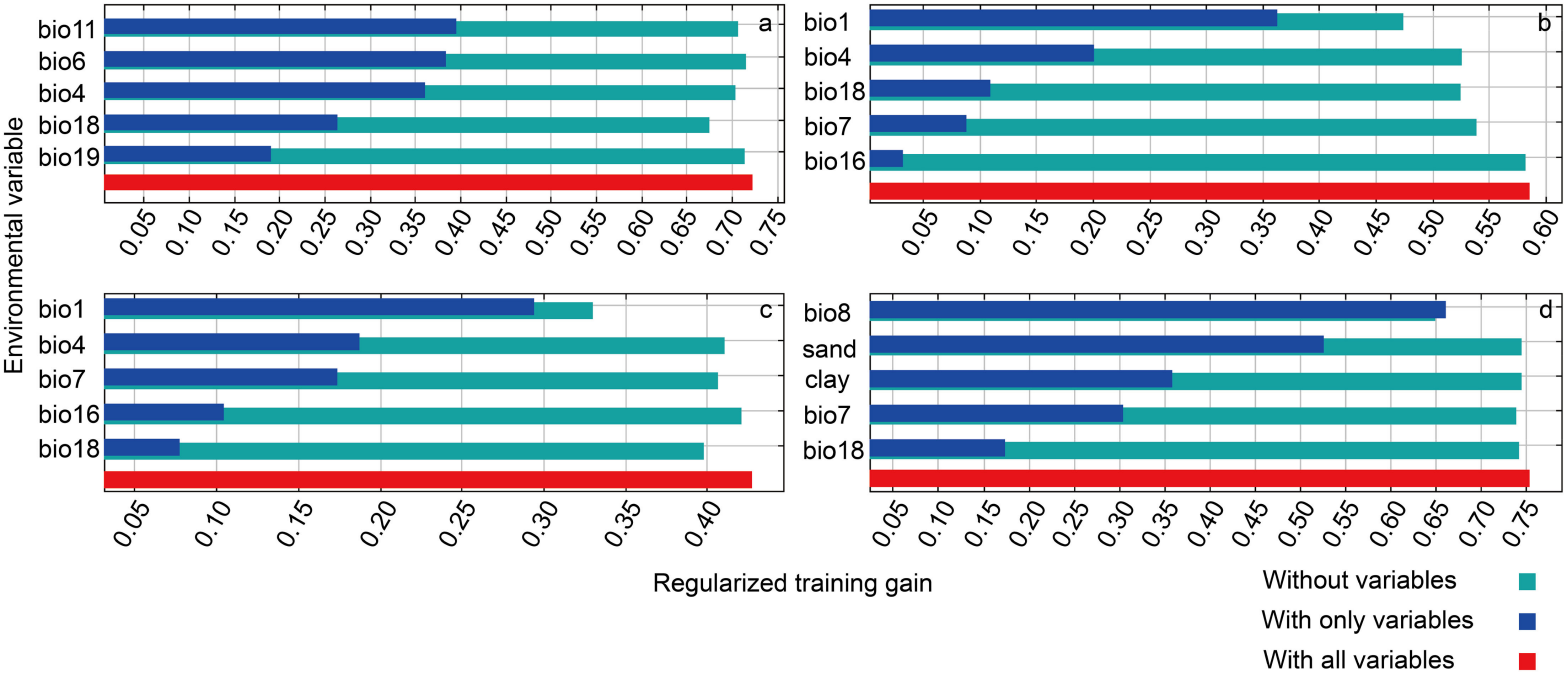
Jackknife test of the importance of top 5 environment variables in MaxEnt. Letters **a–d** in the graph indicates NDVI of high, medium, low, and bare or sparse vegetation, respectively.

The results of MaxEnt model showed that the spatial distribution of PNDVI in Hotan was mainly affected by climate, soil, and groundwater. There are certain differences in the dominant environmental factors corresponding to the PNDVI distribution of different vegetation coverage (**Table 2**). The influential factors with the highest contribution to the simulation of PNDVI spatial distribution (later called as PNDVI distribution) for high vegetation, medium vegetation, low vegetation and bare or sparse vegetation are bio11 (41.9%), boi1 (55.7%), bio1 (27.8%) and bio8 (61.3%), respectively (**Table 2**). In all environmental factors, the contribution of clay is not the highest, but it has significant influence on the simulation of PNDVI distribution with different vegetation coverage, and the contribution rates are 3.3%, 7.7%, 2.5% and 2.4%, respectively (**Table 2**). In addition, the Jackknife module of the maximum entropy model is used to analyze the influence of the weight of the main environmental factors on the simulation of PNDVI distribution in the current climate environment. The results are consistent with the contribution rate of environmental factors (**Figure 4**). For example, when simulating the PNDVI distribution of medium and low vegetation, bio1 provided the highest gains when used independently, indicating that bio1 contained more useful information by themselves than the other variables possessed (**Figures 4B, C**). Overall, the environmental factors that contributed greatly to the simulation of PNDVI spatial distribution in Hotan were mean temperature of the coldest quarter, precipitation of the warmest quarter, precipitation of the coldest quarter, isothermally, proportion of clay particles(<0.002 mm) in the fine earth fraction, annual mean temperature, mean diurnal range, temperature annual range, precipitation of the wettest quarter, mean temperature of the wettest quarter and proportion of sand particles (>0.05 mm) in the fine earth fraction.

### Spatial distribution characteristics of PNDVI in Hotan

**Figure 5:**
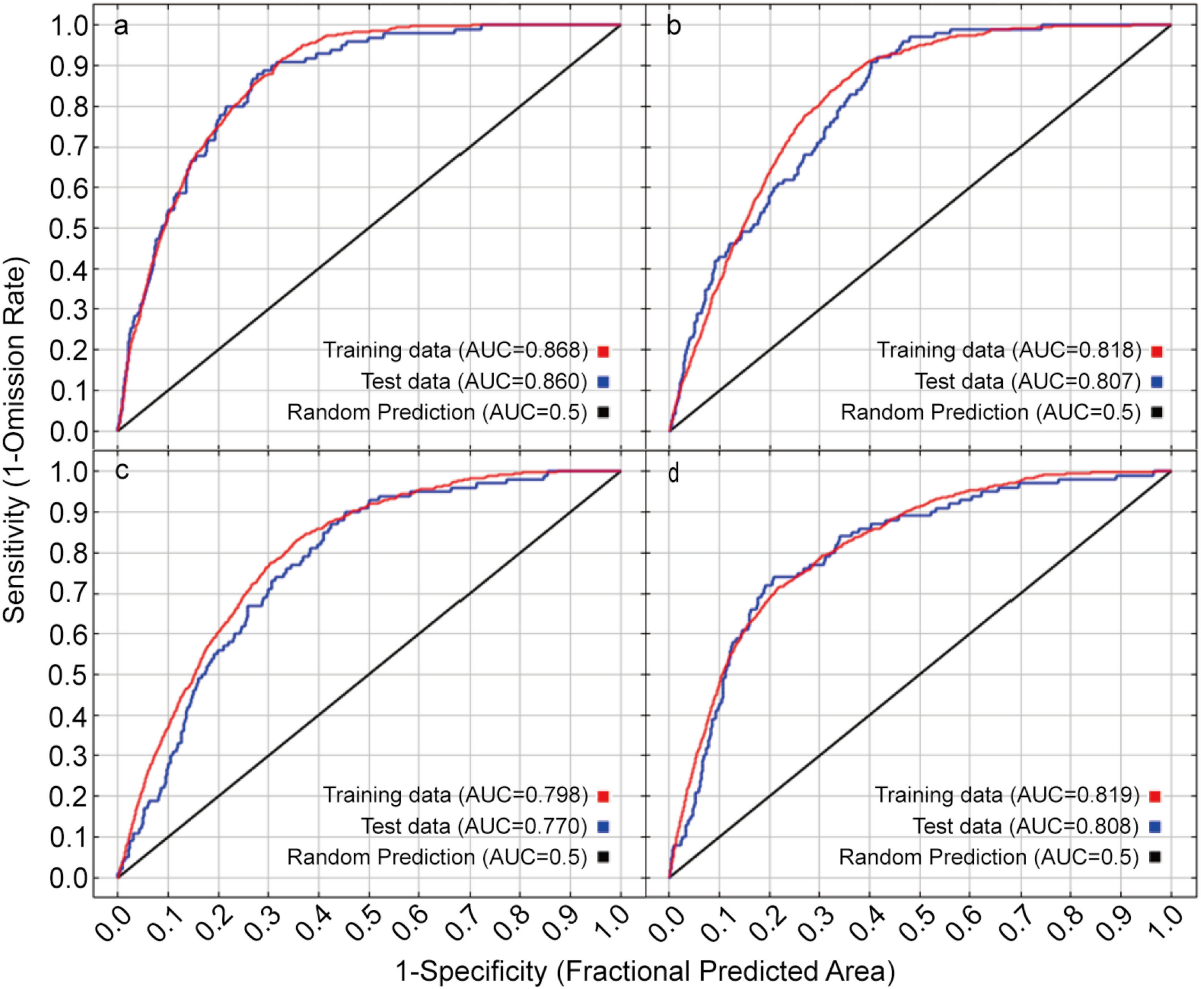
Receiver operating characteristic curve (ROC) of the MaxEnt models. Letter **a–d** in the graph indicates NDVI of high, medium, low, and bare or sparse vegetation respectively.

As a vegetation index, spatial distribution of NDVI also needs to meet the classification criteria of plant survival possibility. Therefore, according to the division standard of the suitable survival possibility of species, the regions with the distribution probability greater than 0.46 were used as the simulation results of PNDVI distribution (Guo et al., 2019). In addition, when the PNDVI distribution areas representing different degrees of vegetation coverage regions overlap, the vegetation coverage grade of PNDVI with the highest distribution probability is selected as the simulation result. In this study, the AUC of MaxEnt model training data for four different degrees of vegetation coverage was more than 75%. The modeling results can be used to predict PNDVI in Hotan (**Figure 5**).

**Figure 6:**
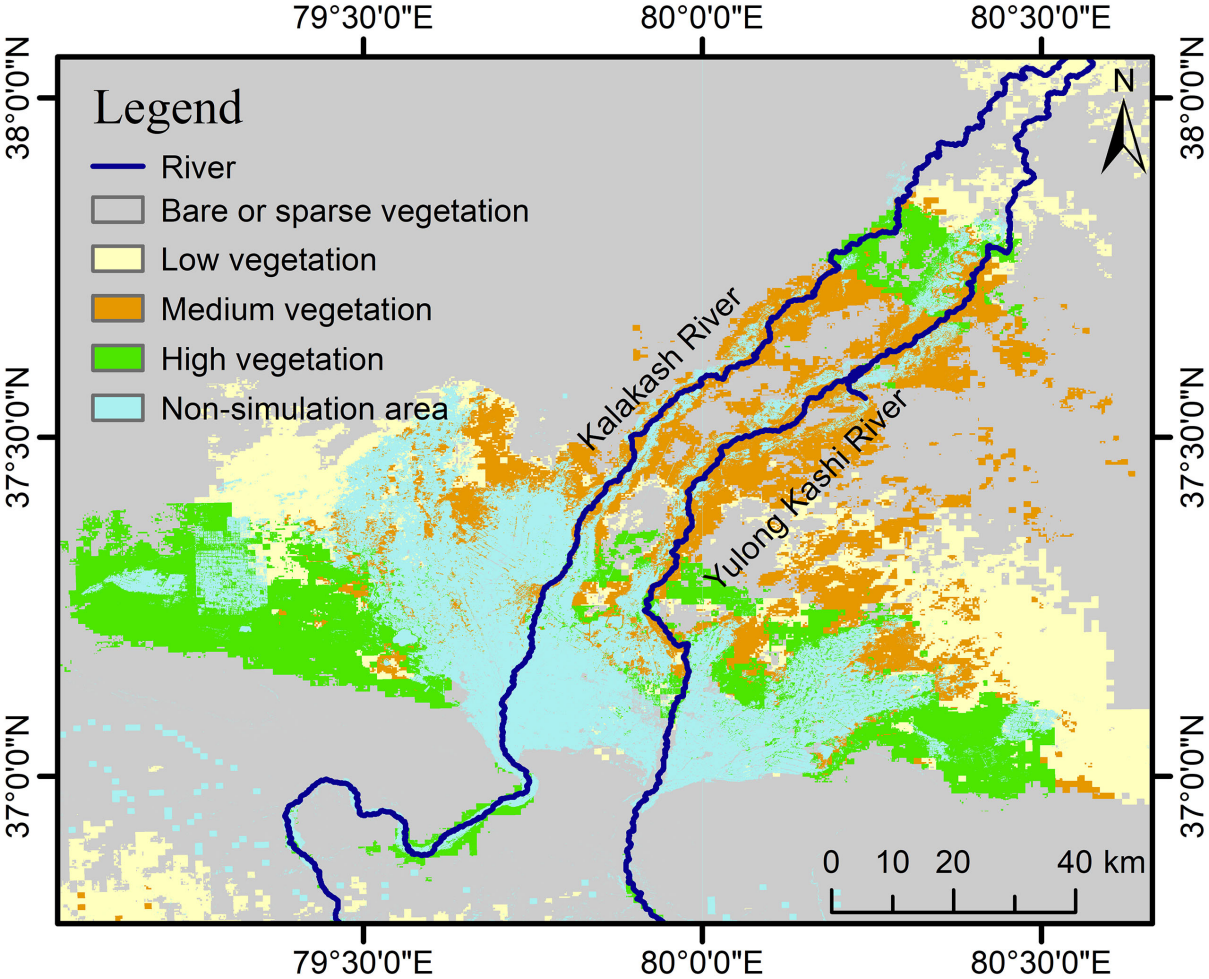
Spatial distribution characteristics of PNDVI in Hotan. Non-simulation area refers to irrigated cropland, impervious surface, and water body.

The simulation results showed that the PNDVI with high vegetation coverage level is mainly distributed in the southwest and southeast of Hotan Oasis and the upstream and downstream of Kalakash River and Yulong Kashi River. The PNDVI of middle vegetation coverage mainly distributed in the midstream and downstream of Kalakash River and Yulong Kashi River. When the representative vegetation coverage is low, PNDVI is distributed in the eastern and western parts of the Hotan Oasis and the intersection area where Kalakash River and Yulong Kashi River combined into the Hotan River. PNDVI with extremely low vegetation coverage is mainly distributed in desert or Gobi area outside the oasis. Overall, the PNDVI value of vegetation showed a decreasing trend from south to north, and the vegetation distribution gradually contracted to the vicinity of the river (**Figure 6**).

**Table 3**, represents the area of ANDVI and PNDVI with different vegetation cover levels. PNDVI with different vegetation covers accounted for 6.8%, 7.26%, 9.17% and 66.95% of the total study area, respectively. Compared with ANDVI, vegetation coverage area increased by 10.20%. In addition, the results of PNDVI simulation showed that the proportion of low, medium, and high vegetation coverage is higher than ANDVI, while the bare or sparse vegetation coverage is less than ANDVI. It can be seen that the vegetation in Hotan still has certain growth potential and can increase the vegetation with different coverage levels by 2192.66 km^2^.

**Table 3:** The area and percentage of ANDVI and PNDVI with different vegetation coverage level.

Vegetation coverage level	ANDVI		PNDVI	
	Area (km^2^)	Percentage (%)	Area (km^2^)	Percentage (%)
High vegetation	44.16	0.20	1471.02	6.80
Medium vegetation	872.98	4.02	1571.026	7.26
Low vegetation	1915.28	8.81	1983.03	9.17
Bare or sparse vegetation	16784.69	77.20	14478.19	66.95

### Analysis of desertification combating needs on PNDVI

**Figure 7:**
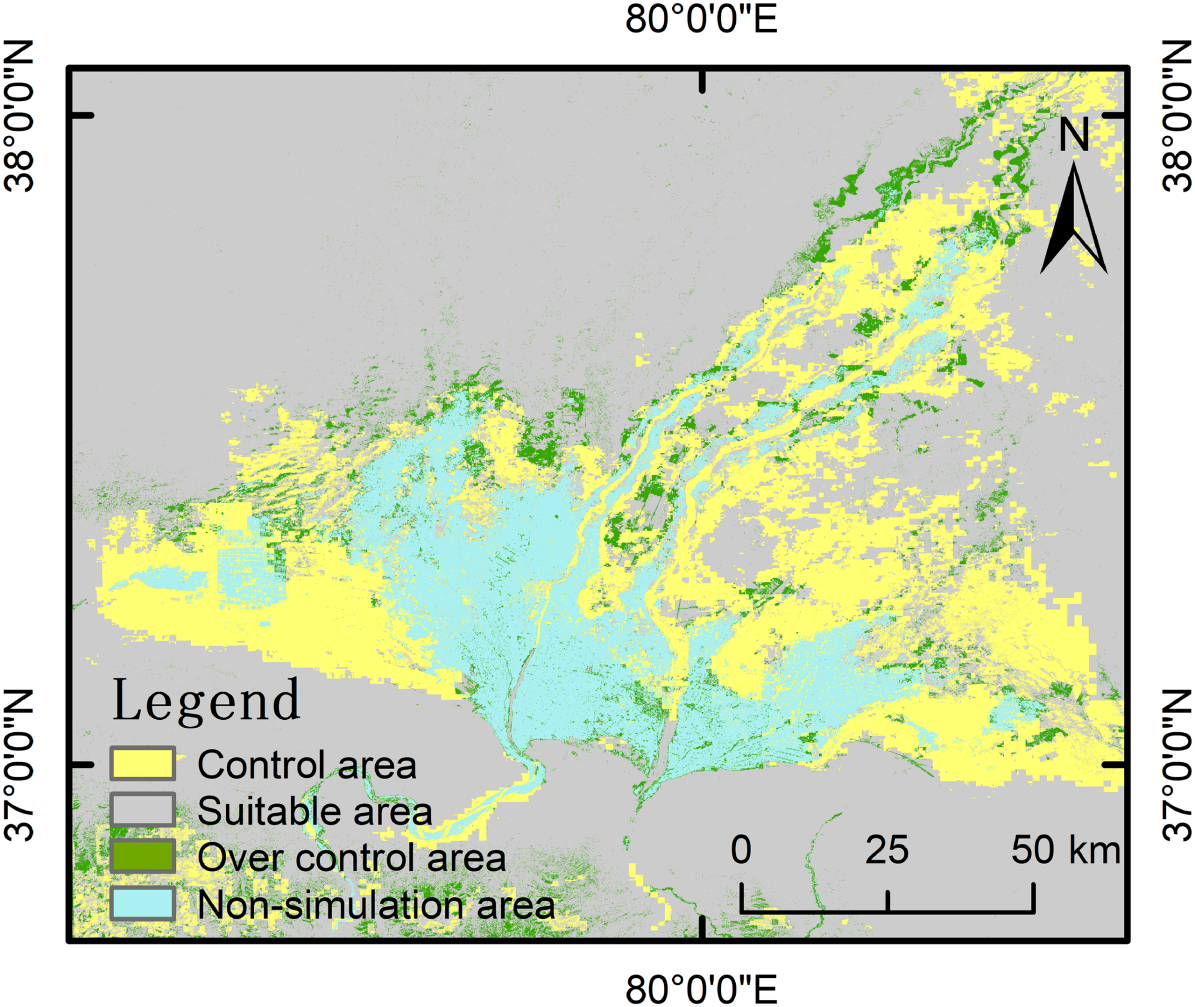
Spatial distribution of desertification combating areas. Control area is the area where desertification control is required; Suitable area is where the vegetation growth has reached the maximum growth potential allowed by the environment; Over contral area is where the vegetation growth exceeds the maximum growth potential allowed by the environment; Non-simulation area refers to irrigated cropland, impervious surface, and water body.

The desertification combating needs of Hotan was analyzed by comparing PNDVI with the ANDVI, and the study area was divided into control areas (PNDVI-ANDVI > 0, the coverage of potential natural vegetation is higher than that of actual vegetation), suitable areas (PNDVI-ANDVI = 0, the coverage of potential natural vegetation is equal to actual vegetation) and over control areas (PNDVI-ANDVI < 0, the coverage of potential natural vegetation is lower than that of actual vegetation). The results showed that PNDVI is generally higher than ANDVI without considering cultivated land, impervious surface, and water bodies. Therefore, Hotan Oasis is still facing serious desertification threats, and desertification combating areas are mainly distributed in the ecotone of desert and oasis. The desertification zones in Hotan are mainly comprised of desert-oasis ecotone, accounting for 18.04% (3902.01 km^2^) of the total area; the over control areas are scattered in the periphery of the irrigated cropland area and the mountainous areas in the southern oasis, accounting for 4.10% (887.13 km^2^) of the study area; and the suitable areas for prevention and control are widely distributed in desert areas, accounting for 68.04% (14713.71 km^2^) (**Figure 7**).

**Figure 8:**
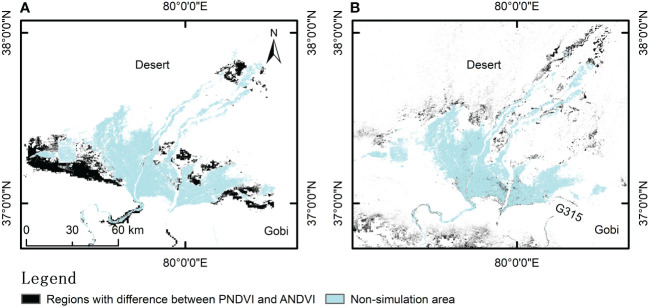
Regions with difference between PNDVI and ANDVI. **(A)** represents the region where PNDVI with high vegetation and ANDVI with bare or sparse vegetation; **(B)** represents the region where PNDVI with bare or sparse vegetation and ANDVI with low vegetation.

In addition, the difference between potential and actual NDVI is mainly manifested in 12 types after grading NDVI according to vegetation cover (**Table 4**). As mentioned above, the regions with PNDVI value higher than ANDVI value are divided into desertification control areas. Therefore, the specific changes in vegetation cover level during desertification can be analyzed by the difference between PNDVI and ANDVI, and effective desertification control measures can be developed on this basis. The desertification control area in Hotan is composed of the region with bare or sparse vegetation which needs to be increased to cover level. Among them, the area (PL-AB) that needs to increase the vegetation coverage to 15% ~ 30% accounts for 5.74% of the study area, whereas the extreme degradation area (PH-AB) where vegetation coverage can reach 60% when the vegetation growth potential is maximum accounts for 4.11% (**Table 4**; **Figure 8A**).

**Table 4:** Composition of control area and over control area.

Control areas			Over control areas		
Type	Area (km^2^)	Percentage (%)	Type	Area (km^2^)	Percentage (%)
PH-AB	889.66	4.11	PB-AL	585.25	2.71
PM-AB	789.16	3.65	PL-AM	151.37	0.70
PL-AB	1241.50	5.74	PB-AM	122.17	0.56
PH-AL	320.22	1.48	PM-AH	13.52	0.06
PM-AL	415.98	1.92	PL-AH	4.86	0.02
PH-AM	245.54	1.14	PB-AH	9.94	0.05

The letters P and A represent PNDVI and ANDVI, respectively; H, M, L, and B represent high vegetation, medium vegetation, low vegetation and bare or sparse vegetation, respectively. Letter combinations represent potential or actual NDVI under different vegetation cover levels.

There are also some other areas where ANDVI is higher than PNDVI. The over control area is mainly characterized by PB-AL which is distributed in the region with actual vegetation coverage between 15% ~ 30% but without vegetation growth potential. PB-AL, accounts for 2.71% of the study area, is distributed in the desert and Gobi areas around Hotan oasis, such as the National Highway G315 (**Table 4**; **Figure 8B**).

## Discussion

Accurate assessment and monitoring of desertification is an important pillar for sustainable development as it informs about the degradation processes, and thus appropriate preventive measures can be initiated in time (Abuzaid and Abdelatif, 2022). In the present study, PNDVI was introduced to determine desertification combat needs in Hotan oasis through the identification of desert-prone areas based on vegetation potential of the oasis. The results revealed that AUC value of the model for the relationship between PNDVI spatial distribution and environmental factors with different vegetation coverage levels was above 0.75, indicating that the MaxEnt model is suitable for simulating the potential distribution of NDVI in the region. It shows that the model is reliable and can effectively be used to estimate vegetation distribution (Qin et al., 2020). Screening of environmental factors revealed that the most influential factors with the highest contribution to the PNDVI distribution for high, medium, low, and bare or sparse vegetation were bio11, bio1, bio1 and bio8, respectively. Particularly, temperature-related factors (mean temperature of the coldest quarter and annual mean temperature) showed pronounced contributions towards vegetation cover distribution. Temperature plays a crucial role in regulating plant photosynthesis and affects plant growth and reproduction *via* light intensity (Zhang et al., 2019). As future temperature and precipitation in Xinjiang are predicted to increase (Du et al., 2021), it may somewhat facilitate desertification combating in Hotan. Furthermore, variation of climate from warm-dry to warm-wet has increased vegetation in Xinjiang.

The simulation results showed that the vegetation coverage represented by PNDVI in Hotan oasis decreases from south to north. With the deepening of the desert, the distribution range of vegetation gradually concentrated near the river. The comparison of PNDVI and ANDVI found that the distribution area of PNDVI with vegetation coverage greater than 15% in the study area increased by 2192.66 km^2^. Our findings are supported by Cai et al. (2022), who reported that the natural vegetation in the desert oasis transitional zone in Hotan still has high growth potential. However, contrary to our findings, Pan and Xu (2020) reported that the vegetation coverage in Hotan is less than 60%. Therefore, the simulation results of this study may be overestimated. The difference in PNDVI simulation results could be attributed to the selection of model, the accuracy of meteorological and soil data, and lack of groundwater data around oasis and desert areas.

PNDVI-based analysis for desertification control needs in Hotan indicated that 18.04% of Hotan region is under serious desertification threats because vegetation cover is not reaching the potential state and needs desertification control measures. In contrast, Nuermaimaiti and Wang (2020) showed that 92.5% of the Hotan area is suffering varying degrees of desertification. Such a large difference in desertification areas appeared because in the current study, we did not consider areas of intense human activities such as farmland and impervious surface in the research process. On the other hand, considering that the nature of desertification is a process of land degradation, this paper uses PNDVI as a benchmark to determine the occurrence area of desertification, rather than a single vegetation coverage to evaluate desertification. Moreover, an 853.65 km^2^ area in the study region also indicated a trend of desertification reversal. Our results are in line with Cai et al. (2022), who reported a significant reversal of desertification within the Hotan oasis, as well as they also proposed PNPP as a benchmark for desertification research in Central Asia as we have used PNDVI as a benchmark for desertification assessment.

The results of this study showed that the precise identification of areas requiring desertification control can be achieved by comparing the PNDVI with the ANDVI. As Paruelo and Lauenroth (1995) proposed that the potential functioning map of the vegetation, like PNDVI, could be compared with actual functioning maps and can be used to monitor the relationship between land cover changes related to human use at the regional scale. Furthermore, Pan and Xu (2020) implied that the spatial simulation of PNDVI and PNPP can separate the direct impact of human activities on natural ecosystems from the impact of climate change and quantify the difference between actual and potential ecological conditions under external pressure. Therefore, the idea of considering biophysical characteristics of potential vegetation as a benchmark to combating desertification is more relevant to the current demand for desertification control aimed at improving the ability of various ecosystems and ensuring benefits of sustainable development (Bastin et al., 2019). Since, this paper highlights the critical role of PNDVI in desertification control research, but the contribution of some relevant indicators describing ecosystem functions of desertification has been neglected, which is a limitation of this study. Therefore, future studies should consider more ecological indicators to build a complete desertification assessment system based on this study’s result. It could help to establish a theoretical basis for accurate desertification assessment.

## Conclusion

In the present study an attempt has been made to use PNDVI for desertification assessment and control needs in the Hotan oasis. By combining the distribution point data of natural vegetation with long-term stable NDVI and 24 environmental factors, the MaxEnt model (Average AUC = 0.826) successfully simulated the spatial distribution of PNDVI and evaluated the demand for desertification control in Hotan oasis. In general, PNDVI spatial distribution with different vegetation cover levels was mainly affected by the annual mean temperature (bio 1), mean temperature of the wettest quarter (bio 8), mean temperature of coldest quarter (bio 11), and groundwater level in growing season. However, the key environmental factors and their contribution rates to PNDVI simulation results at different vegetation cover levels are different. PNDVI simulation results show that the distribution of PNDVI with high, medium, and low vegetation cover accounted for 6.80%, 7.26%, and 9.17% of Hotan oasis, respectively. The comparison between PNDVI and ANDVI shows that Hotan oasis desert ecotone appears to be still the main area for desertification control in Hotan oasis (PNDVI>ANDVI). Most regions of deserts and Gobi have not experienced desertification (PNDVI=ANDVI). In addition, some vegetation in the study area may have excessive growth (PNDVI < ANDVI) due to human activity, such as shelter forest construction and agricultural irrigation. Without considering the actual functions of these vegetation, we believe that excessive prevention and control exist in these areas that do not follow the potential development laws of vegetation. The PNDVI spatial distribution method has provided substantial information regarding assessment and desertification combating needs in Hotan oasis. Therefore, it could be employed as a robust baseline for desertification assessment from regional to global scale, thereby strengthening the efforts to halt desertification.

## Data availability statement

The original contributions presented in the study are included in the article/supplementary material. Further inquiries can be directed to the corresponding author.

## Author contributions

Investigation and data curation: LZ, JQ, QL, ZA, YL. Writing - original draft: LZ and JQ. Conceptualization, writing - review and editing, supervision: DG and ZQ. All authors contributed to the article and approved the submitted version.
